# Acute Appendicitis Associated With Kawasaki Disease: Case Report and Review of the Literature

**DOI:** 10.7759/cureus.18997

**Published:** 2021-10-23

**Authors:** Kaku Kuroda, Morgan Stottlemyre

**Affiliations:** 1 Family Medicine, State University of New York (SUNY) Upstate Medical University, Syracuse, USA; 2 Pediatrics, United States Naval Hospital Okinawa, Ginowan, JPN; 3 Pediatrics, Washington University School of Medicine, St. Louis, USA

**Keywords:** kawasaki disease (kd), acute appendicitis, acute abdomen, persistent fever, mucositis

## Abstract

Acute appendicitis is a rare complication of Kawasaki disease in the setting of the absence of the severe acute respiratory syndrome coronavirus 2 (SARS-CoV-2). We experienced a rare case of acute appendicitis associated with Kawasaki disease. The patient is a six-year-old male who was brought to the emergency department by his mother with a pruritic rash, nausea, vomiting, and abdominal pain. Given fever, tenderness in the right lower quadrant on physical examination, leukocytosis with bandemia, and a non-compressible and dilated appendix on ultrasound, he was diagnosed with acute appendicitis and was treated with a laparoscopic appendectomy. He developed persistent fevers after surgery with new lip swelling, mucositis, and bilateral conjunctival injection. Kawasaki disease was suspected and intravenous gammaglobulin and aspirin were administrated. He made a full recovery. This case suggests that careful examination is needed for accurate diagnosis, especially in patients with postoperative persistent fever without signs of intra-abdominal complications. We performed a PubMed literature search and reviewed eight cases of appendicitis associated with Kawasaki disease. Of note, this case was seen in 2018 before the SARS-CoV-2 pandemic and the description of multisystem inflammatory syndrome in children (MIS-C).

## Introduction

Kawasaki disease is a systemic, inflammatory illness that affects medium-sized arteries in children [1]. As a result of this widespread inflammation, the clinical features of Kawasaki disease are various, including fever, conjunctivitis, erythema of the lips and oral mucosa, rash, extremity changes, and cervical lymphadenopathy. Although abdominal symptoms are a common feature of Kawasaki disease [2], acute appendicitis is a rare complication in the setting of the absence of severe acute respiratory syndrome coronavirus 2 (SARS-CoV-2). Given the difficulty in diagnosing Kawasaki disease and its complications, we believe that it is important to discuss unusual presentations. We herein report the rare case of acute appendicitis associated with Kawasaki disease and a literature review about this rare condition.

## Case presentation

**Figure 1:**
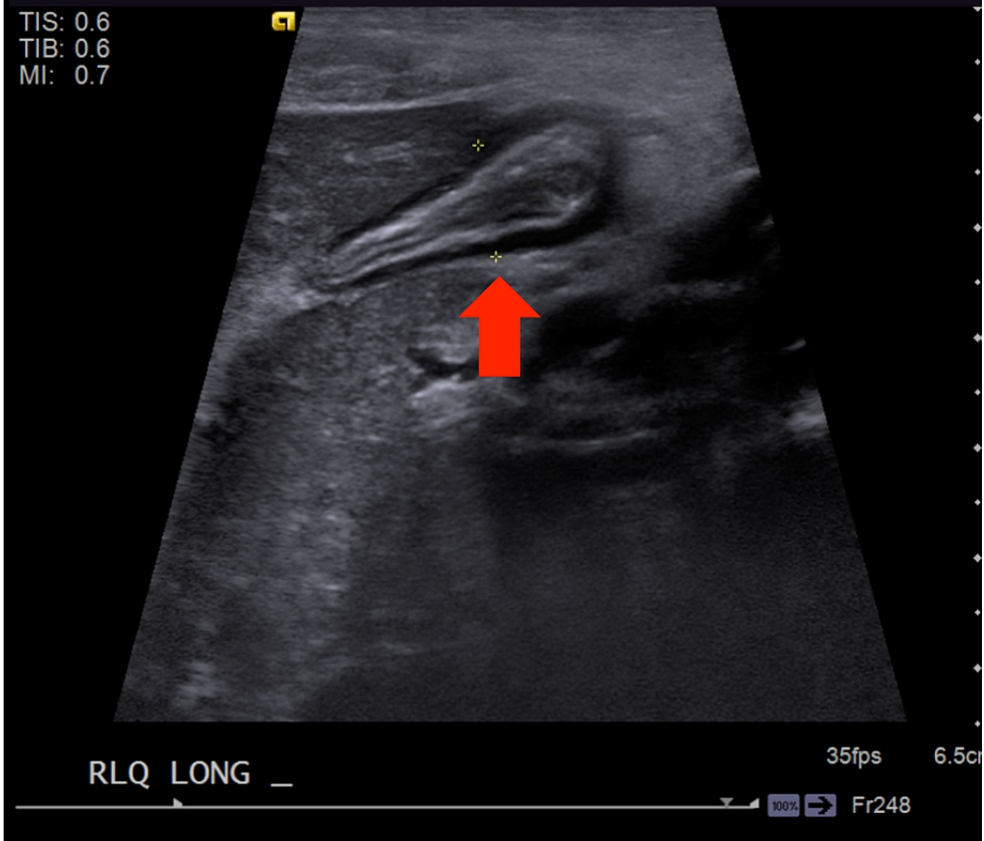
Sonographic examination of the right lower quadrant The appendix is identified within the right lower quadrant (red arrow). The finding is non-compressible and measures approximately 1 cm in diameter. Additionally, there is edema of the wall of this structure.

A six-year-old male was brought to the emergency department (ED) by his mother with a pruritic rash, nausea, vomiting, and abdominal pain. This case occurred in 2018 before the emergence of the SARS-CoV-2 virus. Abdominal pain, nausea, anorexia, and emesis started around noon on the day of the visit as well as fevers and chills. In addition to his abdominal symptoms, he presented with a rash that had developed that morning. The patient’s history was significant for allergic rhinitis, eczema, and peanut allergy. His initial vitals were remarkable for fever of 101.6 (38.7℃), and physical examination demonstrated tenderness in the right lower quadrant and maculopapular rash over the upper and lower extremities. Laboratory data showed a white blood cell count of 13,700/mm^3^, with neutrophils of 91% and C-reactive protein of 1.41 mg/dL. Ultrasound revealed a non-compressible appendix, measured 10 mm in diameter, with edema of the wall of this structure (Figure 1).

Acute appendicitis was suspected, and the patient was taken for laparoscopic appendectomy the next day. Intraoperative findings showed an infected appendix with pelvic fluid and pathology demonstrated focal inflammation with neutrophil and eosinophil invasion, which was consistent with early acute appendicitis. Given persistent postoperative fever without signs of abscess or intra-abdominal complications in the setting of new onset lip swelling, mucositis, and bilateral conjunctival injection, the patient was subsequently suspected of incomplete Kawasaki disease. Intravenous gammaglobulin (IVIg) and aspirin were administrated on hospital day 12, which was day 9 of consecutive fever. The patient was afebrile within 24 hours of treatment. An echocardiogram was obtained, which showed no abnormal findings including a coronary aneurysm. He made a full recovery and the further course was uncomplicated at a follow-up.

## Discussion

We experienced a case of acute appendicitis associated with incomplete Kawasaki disease. This patient was seen before COVID-19 emerged. However, in this era of the COVID-19 pandemic, a multisystem inflammatory syndrome in children (MIS-C) should be suspected. MIS-C is a relatively rare complication of COVID-19 in children that can mimic Kawasaki disease or toxic shock syndrome. It is thought to derive from abnormal cytokine release and macrophage activation, but the exact pathophysiology is unknown. It is reported that approximately 40% of patients with MIS-C met the criteria for complete or incomplete Kawasaki disease [3], and there is a wide range of overlap between MIS-C and Kawasaki disease. However, Kawasaki disease tends to affect younger children while MIS-C affects children older than 5 years [3-4]. With regards to clinical presentation, gastrointestinal symptoms, including abdominal pain are predominant in patients with MIS-C [5]. It was reported that abdominal pain was present in 285/783 patients with MIS-C (36 percent) as a clinical symptom [6]. The presentation has been well-described to mimic acute appendicitis [7]. Several cases of children with appendicitis possibly associated with COVID-19 infection have been reported [8-9].

Gastrointestinal symptoms, including diarrhea, vomiting, and abdominal pain, are common in Kawasaki disease [2]. Furthermore, according to a previous study, 4.6 percent of patients presented with an acute surgical abdomen [10].

We did a literature review about appendicitis as a complication of Kawasaki disease. We performed a PubMed literature search using the search terms "appendicitis" and "Kawasaki disease". Since this case was seen before COVID-19 emerged, we did not include “COVID-19” or “MIS-C”. We found eight cases of appendicitis associated with Kawasaki disease [10-14]. We reviewed those cases in terms of age/gender, initial symptoms, laboratory data, clinical course, treatment and response, intraoperative findings, and pathology of the appendix (Table 1).

**Table 1:** Details of eight cases of appendicitis associated with Kawasaki disease *Abbreviations: WBC: white blood cell; CRP: C reactive protein

No	Age/gender	Presenting symptoms	Laboratory data	Intraoperative findings	Pathology	Reference
1	3/male	72-hour history of periumbilical abdominal pain, nausea, vomiting, diarrhea, and fever	Leukocytosis with bandemia, elevated CRP, and acidosis	Free pelvic fluid and an injected appendix with serosal changes but minimal thickening	Focal intramuscular inflammation with neutron invasion	Garnett GM, et al. [11]
2	7/female	4-day history of abdominal pain, emesis, diarrhea, fever, and bilateral non-exudative conjunctivitis	A markedly elevated CRP level and white blood cell (WBC) count	Suppurative appendicitis, purulent pelvic fluid, and uniform dilation of the entire small bowel	Mild neutrophilic and eosinophilic appendicitis	Garnett GM, et al. [11]
3	6/male	4-day history of lower abdominal pain, nausea, vomiting, and fever of 38℃	WBC of 21,300/mm3, CRP of 11.0 mg/dl	Acute phlegmonous appendicitis	No details	Chiba T. [12]
4	4/male	3-day history of fever of 38°C and severe colicky abdominal pain in the right lower quadrant	WBC of 12,600 /mm3, CRP of 7.0 mg/di	An enlarged, firm, inflamed appendix	Moderate inflammatory changes with edema.	Chiba T. [12]
5	5/male	A sudden onset of high fever (39–40°C) and acute abdominal pain	WBC 21600/mm3 (neutrophils 90%), CRP 6.9 mg/dL, ESR 90 mm/h, hemoglobin 11.2 g/dL, platelet count 320,000/mm3, ALT 74 U/L, AST 57 U/L, and GGT 106.	Inflammation of the peritoneum and appendix	An acute transmural inflammation with diffuse arteritis.	Zulian F, et al. [10]
6	3/?	Fever (remittent, high-spiking 37.5-39 °C, 2-3 spikes/day, persisting for two weeks), right lower quadrant abdominal pain	No details	No details	Appendicular vasculitis with peritoneal inflammation and serous secretion	Maggio MC, et al. [14]
7,8	?	Acute abdominal pain	No details	No details	The final histological diagnosis of appendicitis	Velez-Tirado N, et al. [13]

Seven out of eight cases were described to have histologically proven appendicitis. In three of these seven cases, pathology showed inflammatory changes in the mucosa or muscle of the appendix. Two of them were found to have findings of vasculitis in the appendix. Postoperative courses were complicated by persistent fever and new inflammatory symptoms, such as conjunctival or skin changes in all cases, and the diagnosis of Kawasaki disease was made after surgery (Table 2).

**Table 2:** Postoperative course and treatment of eight cases of appendicitis associated with Kawasaki disease *Abbreviations: IVIg: intravenous gammaglobulin

No	Age/gender	Postoperative course	Treatment	Reference
1	3/male	Anorexia, persistent abdominal pain, diarrhea, transaminitis, and high fevers. Peripheral edema, involving the hands, fingers, and feet, conjunctival injection, and dry, cracked lips developed.	IVIg and aspirin with good response	Garnett GM, et al. [11]
2	7/female	Persistent abdominal pain, fever, the development of a raised rash on the arms, trunk, and buttocks, and dry, cracked lips.	IVIg and antiplatelet therapy with good response	Garnett GM, et al. [11]
3	6/male	Fever was down for one day postoperatively, then up again with increasing maculopapular rash, conjunctival ecchymosis, swelling of the lips, and maceration and peeling of the skin of the feet and scrotum.	IVIg with good response	Chiba T. [12]
4	4/male	A maculopapular rash of the trunk, conjunctivitis, cervical adenopathy, and desquamation of the hand was noticed two days after the operation.	IVIg with good response	Chiba T. [12]
5	5/male	A maculopapular rash on the trunk and lower limbs, conjunctivitis, mucositis, and edema of both hands and feet developed	IVIg with good response	Zulian F, et al. [10]
6	3/?	Fever persisted; some days later, he presented conjunctivitis, lips cracking, the elevation of the erythrocyte sedimentation rate, and C-reactive protein, thrombocytosis (715.000). He also developed hands edema and periungual fingers peeling	IVIg and aspirin with good response	Maggio MC, et al. [14]
7,8	?	The Kawasaki disease diagnosis was made after the surgery when they persisted with florid systemic symptoms: high fever, conjunctival hyperemia, generalized rash, upper and lower extremities edema, and desquamation.	IVIg, aspirin, and steroids with good response	Velez-Tirado N, et al. [13]

Five cases met the criteria for classic Kawasaki disease and three cases were consistent with incomplete Kawasaki disease. Regarding treatment, IVIg was effective in all cases. Proposed hypotheses involve the upregulation of VB2 T cells in the blood and jejunal mucosa in Kawasaki disease as well as the role of lymphoid tissue in the appendix as it relates to immune response [15-18].

While Kawasaki disease remains the most likely diagnosis, this case was limited by the location at an overseas U.S. military hospital, where testing for infectious diseases such as *Yersinia enterocolitica* (which can mimic both conditions) [19] was unable to be performed in a timely manner. There are also differential diagnoses with other inflammatory processes such as IgA vasculitis or eosinophilic gastroenteritis. However, the presentation and resolution of fever with IVIg are highly suggestive of Kawasaki disease.

## Conclusions

We presented a case of acute appendicitis associated with incomplete Kawasaki disease. While these two diagnoses do not occur together often, careful examination is needed for an accurate diagnosis to allow for timely management even in the setting of the absence of COVID-19, particularly in patients with postoperative persistent fever without signs of intra-abdominal complications and mucosal or skin changes. Additionally, MIS-C should be considered as a differential diagnosis in this era of the COVID-19 pandemic.
